# *Breathlessness limiting exertion* and under- and unemployment: results from a national, online population survey

**DOI:** 10.1186/s12889-026-28996-5

**Published:** 2026-08-14

**Authors:** Jacob Sandberg, Max Olsson, Sungwon Chang, Slavica Kochovska, Nikki McCaffrey, Joseph Clark, Magnus Ekström, David Currow

**Affiliations:** 1 Department of Health, Blekinge Institute of Technology, Karlskrona, Sweden; 2 Faculty of Medicine, Department of Clinical Sciences Lund, Respiratory Medicine, Allergology and Palliative Medicine, Lund University, Lund, Sweden; 3 Faculty of Health, IMPACCT, University of Technology Sydney, Sydney, NSW Australia; 4 College of Medicine and Public Health, Flinders University, Bedford Park, Adelaide, South Australia Australia; 5 Deakin University, Geelong, VIC Australia; 6 Wolfson Palliative Care Research Centre, Hull York Medical School, University of Hull, Hull, UK

**Keywords:** Breathlessness, Employment, Finances, Financial well-being, Cross-sectional survey

## Abstract

**Background:**

Loss of, or unwanted changes in employment reduces economic wellbeing and is associated with lower quality of life. People living with chronic breathlessness are less likely to participate in the workforce. The influence of breathlessness on change in employment status is unknown. This study investigates associations between self-reported *breathlessness limiting exertion* (hereafter *breathlessness*) and changes in employment status.

**Methods:**

Cross-sectional, online population survey of individuals aged 18–65 years old from Australia. Current and change of employment status, and *breathlessness* (assessed using modified Medical Research Council [mMRC] ≥ 1) were analysed using multivariate logistic regression to establish odds ratio (OR) and 95% confidence intervals (95%CI), adjusted for possible confounding effects.

**Results:**

Eight thousand people were included; 5,730 (72%) were employed and 43% reported *breathlessness*. *Breathlessness* was associated with a change from full-time employment to another employment status: mMRC1 OR: mMRC1 OR: 6.60 (3.4–13.7); mMRC2 OR: 15.7 (7.7–34.2); and mMRC3-4 OR: 31.9 (13.8–75.4); and with being unemployed: mMRC1 OR: 1.20 (1.05–1.4); mMRC2 OR: 1.57 (1.3–1.9); and mMRC3-4 OR: 1.59 (1.1–2.3).

**Conclusion:**

*Breathlessness* is associated with lower rates of employment and a higher probability of reducing work hours or becoming unemployed. The presence of chronic *breathlessness* should lead health professionals to explore the employment (and financial) consequences for the person.

**Supplementary Information:**

The online version contains supplementary material available at 10.1186/s12889-026-28996-5.

## Introduction

In people of working age, workforce participation is associated with a range of positive aspects of wellbeing and health, extending beyond the obvious financial rewards [1, 2]. Having stable employment is crucial for overall health and wellness [3]. In contrast, losing or experiencing unwanted changes in employment status due to illness is associated with higher financial stress and lower quality of life [4]. Chronic cardiorespiratory illness often impacts the ability to work at ones full potential resulting in different stages of *underemployment* [5], (involuntarily working less than full-time) and is also a common reason for premature exit from the workforce resulting in *unemployment*, e.g. not having any employment [6].

*Chronic breathlessness limiting exertion* (hereafter *breathlessness*) is a highly prevalent symptom across populations. It is most often caused by underlying cardiorespiratory diseases such as chronic obstructive pulmonary disease (COPD) or heart failure or by obesity [7–10]. Estimates in high-income countries range from 4 to 10% of the general population [8], and is suspected to be much more common in lower- and middle-income countries (up to 44% of the adult population [11]. Breathlessness is a largely invisible symptom which causes disability and a range of physical, social, financial, sexual and psychological consequences such as poorer quality of life, less ability to care for oneself and higher rate of social isolation [12–16].

A population study conducted in Australia identified that workforce participation was lower among individuals experiencing breathlessness due to multiple conditions, this was especially true for women and increased with age [17]. The inability to stay employed when experiencing breathlessness has large economic implications resulting in a loss in annual income in the range of AU$9.1 billion for full-time work [17]. Underemployment was not included but would further aggravate the cost by individuals reducing working hours to accommodate breathlessness. The magnitude of this impact through potential income foregone suggests that breathlessness has effects on both the personal income and on household income. Increasing breathlessness is also associated with both higher amounts of taking time off from work as well as a poorer performance while at work [7]. More evidence is needed on how employment status changes over time due to breathlessness and the present study extends previous work by examining self-reported employment changes attributed to breathlessness and by including underemployment. Our hypothesis is that breathlessness would be associated with negative consequences on work force participation and work ability.

The primary aim of this study was to explore the relationship between self-reported breathlessness and under- and unemployment among Australian adults of working age.

## Methods

### Study design

This was a cross-sectional, online, population-based survey using the Qualtrics software Copyright ©2021 Qualtrics. Qualtrics and all other Qualtrics products or service names are registered trademarks or trademarks of Qualtrics, Provo, Utah, USA.

Recruitment was provided through a panel of 800,000 maintained by Qualtrics market research, but questionnaire development and sample sizes were at the sole discretion of the researchers without input from Qualtrics market research.

Evidence from population studies is warranted as people with the most severe breathlessness often avoid contact with health services except in extreme circumstances [18] and, by definition, are often housebound [19]. This even limits the use of in-home, face-to-face surveys [20]. As such (and building on previous population-based surveys that are independent of health service contact), this study used an internet survey adjusted to key characteristics of the national population.

Eligibility criterion for the current study was limited to adults of Australian normal working age which is between 18 and 65 years of age. Proficiency in English was needed in order to participate and inclusion was aimed at creating a sample representative of the most recent Australian national census available at the study time (i.e. 2016) [21]. The research team chose study-appropriate goals for age, sex, rurality (metropolitan/non-metropolitan) and state or territory of residence, thereby creating different “cells”. If a respondent fulfilled the criteria for a ‘cell’ that had not reached the expected numbers of responses, the respondent would proceed to the full questionnaire. If that ‘cell’ already had the required number of respondents, the person would be directed away from the survey.

The survey was pilot tested in June 2021 with 110 Qualtrics panel members and no changes were made afterwards. The main data collection period was from mid-July to early August 2021. The complete questionnaire is available online [22].

### Setting

Australia is a high-income country with a population of 27 million people. In August 2024, employment was at 67.0% among people in working age (with a 71.0% participation rate for men and 63.1% for women), unemployment was at 4.1% and under-employment was at 6.4%. (23) Of the 12 million people employed in 2021 (the latest national census), 7 million worked full-time, 4 million worked part-time and 1 million were away from work (holiday/sickness/other reason) [23]. Of the employed, 13.8% were self-employed (with men more likely to be self-employed than women; 65.5% vs. 34.5%) and 4.9% were aged 65 years or older [24]. Retirement income derives from superannuation (which can be accessed when aged ≥ 60 years and retired or ≥ 65 years and still working) and Government Age Pension (which can be accessed when ≥ 67 years of age and is means-tested). Employees are entitled to paid leave taken as either sick leave (for personal illness or injury) or carer’s leave (when caring for family members because of illness or injury), with allocated 10 days per year for full-time employees and pro-rata for part-time employees [22]. Income support for people who are unemployed is available, including for those living with disabilities. The health system is underpinned by a universal health insurance scheme (Medicare), complemented with out-of-pocket co-payments and private health insurance.

### Assessments

*Demographic characteristics* included age, sex, state/territory of residence, rurality (calculated using postcodes and the “Accessibility and Remoteness Index of Australia” [25], height, weight and smoking habits (current, former or never smoker). Height and weight were used to calculate body mass index (BMI).

*Current employment status* was self-reported by selecting one of the pre-defined employment categories (full-time, part-time, casual, self-employed, retired, unemployed, paid leave, unpaid leave, or other).

*Breathlessness* was assessed using the modified Medical Research Council (mMRC) breathlessness scale. The mMRC is a five-point ordinal measure of exertional breathlessness ranging from (‘I only get breathless with strenuous exercise’) to 4 (‘I am too breathless to leave the house or I am breathless when dressing’) [26]. Individuals reporting exertional breathlessness (mMRC ≥ 1) were additionally asked about their perceived underlying cause of breathlessness using the question: “What do you understand is causing your shortness of breath?”. Respondents were asked to identify the primary perceived cause by selecting one response only from predefined categories: lungs, heart, disorder of nerves or muscles, cancer, other (free-text), don’t know, or prefer not to say, with illustrative examples provided for the disease-specific categories. Finally, individuals reporting exertional breathlessness were asked whether they perceived that their breathlessness had affected their employment status using the question: “Has your employment status been affected because of your breathlessness?”. Response options were ‘Yes’ (with follow-up questions), No’, Prefer not to say’, and ‘Not applicable’. Respondents responding ‘Yes’ were subsequently asked to report their previous employment status by selecting one of the following categories: employed full-time, employed part-time, employed casual, self-employed, other (free-text), prefer not to say, or not applicable.

Using the self-reported responses of current and previous employment status, we analysed change from “employed full-time” to any other employment status within the employed group (and not unemployed) among those responding that breathlessness had affected their employment status.

Change from employment to unemployment was explored by grouping responses into categories “employed”, “unemployed” or “underemployed”. *Employed* included responses “employed full-time”, “employed part-time”, “employed casual”, “on paid leave” and “self-employed”. The category *unemployed* included responses: “on unpaid leave”, “retired, or “other”. Responses that were: “prefer not to say” or “not applicable” were considered as missing values. The category *underemployment* includes all non-full-time employment categories (but not unemployed). Details about the questions about employment status and the categorization are presented in Supplementary Table S1.

### Statistical analyses

Baseline characteristics were summarized usings means and medians with corresponding standard deviations (SD) or interquartile ranges (IQR) and then compared by employment status (employed or unemployed), using t-test or chi^2^. Respondents experiencing that their breathlessness is affecting their employment among different mMRC grades were evaluated using chi^2^. Significance was defined as two-sided *p* < 0.05.

Associations between employment status and *breathlessness* severity as well as perceived impact from breathlessness on employment status were explored by calculating the odds ratio (OR) for each mMRC grade using logistic regression with mMRC 0 as the reference value. Possible confounding effects from age, sex, BMI and rurality were controlled after first performing directed acrylic graph (DAG) using dagitty.net, a tool for systematic identification of possible confounders [27].

Logistic regression was also used to assess the relationship between breathlessness and underemployment by calculating the odds ratio for changing from employed full-time to any other level of employment for and finally also for change from any employed status to unemployed status.

Analyses were carried out using the software R, version 4.4.0 (R Foundation for Statistical Computing, Austria). No data were imputed, and missing data were assumed to be missing at random.

## Results

### Population

A total of 10,033 individuals responded to the questionnaire, of whom 8,000 individuals were of working age (< 65) and therefore included in this study. Mean age was 39 (SD 14) and 43% were males and 56% females. Mean BMI was 26.76 (SD 7.48) and 50% were never smokers. Most (*n* = 5,730; 72%) were categorized as employed and 2,115 (27%) as unemployed (1% missing; Table 1).

**Table 1:** – Particpants characteristics

		Total	Employed	Unemployed	*p*-value
		8000	5730 (72%)	2115 (28%)	
Age	mean (SD)	38.62 (13.9)	37.21 (12.9)	42.7 (15.6)	< 0.001
	median (IQR)	37 (24)	36 (21)	44 (30)	
Sex	Female (%)	4488 (56)	3054 (53)	1323 (63%)	< 0.001
	Male (%)	3449 (43)	2634 (46)	778 (36.8)	
Body mass index (BMI)	mean (SD)	26.76 (7.5)	26.2 (7.2)	28.5 (8.1)	< 0.001
	median (IQR)	25.4 (7.4)	24.8 (6.9)	27.3 (9.5)	
Smoking status (%)					< 0.001
Current		2037 (26)	1516 (27)	492 (23)	
Former		1880 (24)	1292 (23)	567 (27)	
Never		3966 (50)	2852 (50)	1031 (49)	
Prefer not to say		117 (2)	70 (1)	25 (1)	
mMRC breathlessness rating (%)					
0		4593 (57)	3376 (59)	1132 (54)	< 0.001
1		2501 (31)	1757 (31)	703 (33)	
2		685 (9)	459 (8)	208 (10)	
3–4		221 (3)	138 (2)	72 (4)	
Employment affected by breathlessness (%)		856 (11)	768 (13)	88 (4)	< 0.001
Cause of breathlessness, self report (%)					
Lungs (e.g. emphysema, smoking, asbestosis, work related, asthma)			903 (16)	333 (16)	
Heart (e.g. ischaemic heart disease, valve disease, abnormal heart rhythms)			314 (6)	63 (3)	
Disorder of nerves or muscles (e.g. motor neurone disease, multiple sclerosis)			124 (2)	33 (2)	
Cancer			25 (1)	13 (1)	
Other			334 (6)	202 (10)	
Don’t know			592 (10)	320 (15)	
Prefer not to say			62 (1)	19 (1)	

*mMRC* modified Medical Research Council breathlessness scale, *SD* standard deviation, *IQR* interquartile range

Differences were analysed with t-tests (for continuous variables) or chi-2 tests (categorical variables). Employed = “Employed full-time”, “Employed part-time”, “Employed casual”, “Self-employed”, “On paid leave”. Unemployed = “Unemployed”, “On unpaid leave”, “Retired”, or “Other”. *Only answered by respondents with mMRC ≥ 1.

The unemployed group was slightly older (mean age 42 [SD 15.6]) and included a higher proportion of females (63%) than the employed group (mean age 37 [SD 13], 53% females. Both groups had similar smoking status, but the unemployed group had a higher mean BMI of 28.5 (SD 8.1) than the employed group (26.2 [SD 7.2]). (Table 1)

Clinically important breathlessnes*s* (mMRC ≥ 2) was higher among unemployed (14%) than among employed (10%) people (*p* < 0.001). The experience that breathlessness is affecting employment was increasing along mMRC grade (mMRC 0: 7%; mMRC 1: 14%; mMRC 2: 32%; mMRC 3–4: 36%, χ = 466; p = < 0.001). The most commonly stated underlying conditions were conditions related to the lungs (16%) or unknown to the respondent (15%). (Table 1) The distribution of mMRC grades by employment status is presented in Supplementary Table S2.

### Breathlessness and employment

**Fig. 1:**
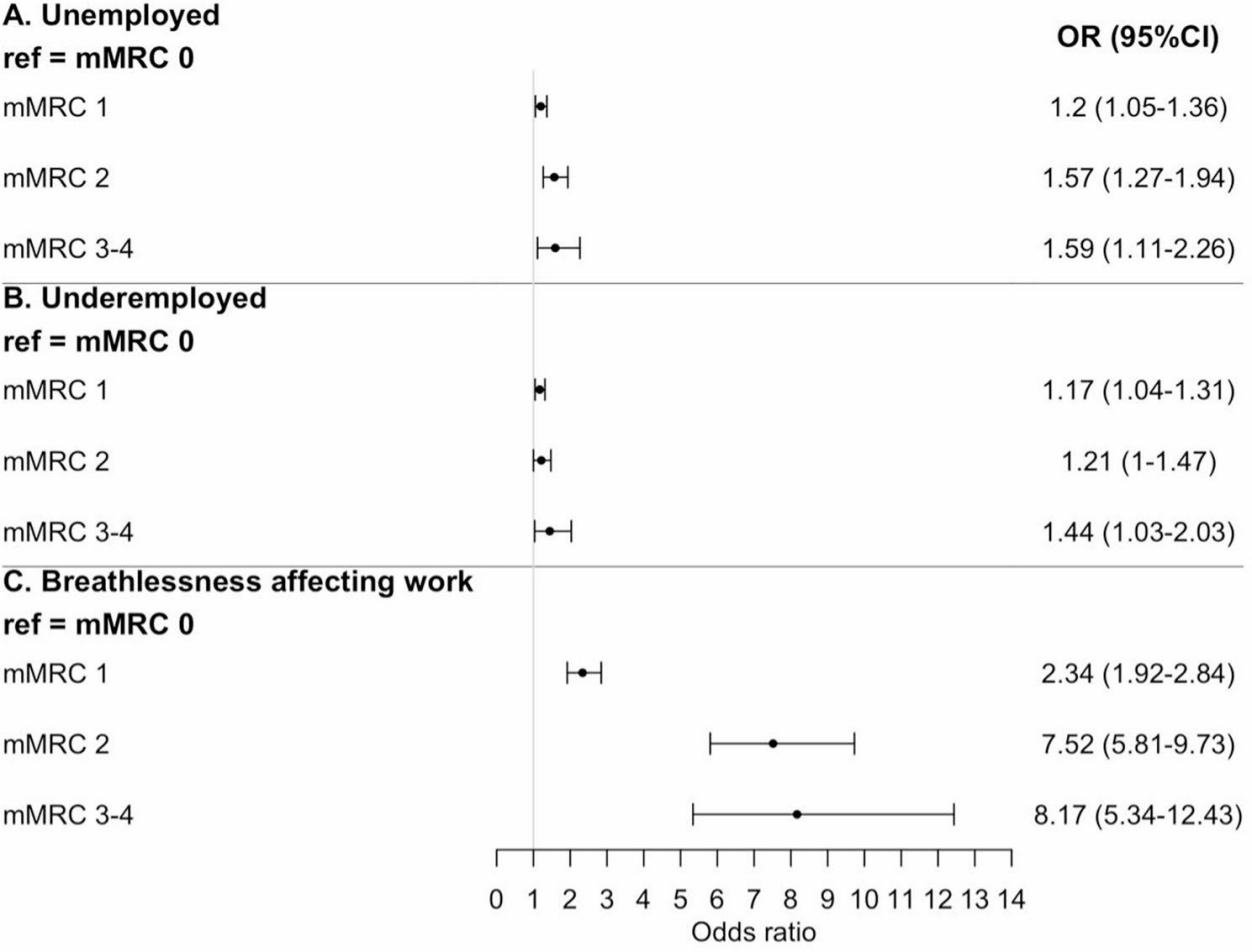
– Association between breathlessness severity, employment status, and breathlessness affecting work among Australians aged under 65. *Logistic regression adjusted for age*,* sex*,* BMI and rurality. *Unemployed= “On unpaid leave”, “Retired”, or “Other”.Employed= “Employed full-time”, “Employed part-time”, “Employed casual”, “Self-employed”, “On paid leave”.Underemployed= being employed but not answering “Employed full-time”. In the analysis of “breathlessness affecting work people that belong to the categories “Employed full-time”, “Employed part-time”, “Employed casual”, “Self-employed” were included. Abbreviations: CI = confidence intervals; mMRC = modified Medical Research council breathlessness scale; OR = Odds ratio

Breathlessness was associated with higher odds of being unemployed: for mMRC1 OR: 1.20; (95% CI) 1.05–1.36; mMRC2 OR: 1.57; 1.27–1.94; and mMRC3-4 OR: 1.59; 1.11–2.26, Fig. 1a. Breathlessness was also associated with being underemployed for: mMRC1 OR: 1.17; (95% CI) 1.04–1.31; mMRC2 OR: 1.21; 1.0–1.47; and mMRC3-4 OR: 1.44; 1.03–2.03, Fig. 1b.

Higher mMRC grade was associated with self-reported affect from breathlessness on employment status: mMRC1 OR: 2.34; 1.92–2.84; mMRC2 OR: 7.52; 5.81–9.73, and mMRC3-4 OR: 8.17; 5.34–12.43, Fig. 1c.

**Fig. 2:**
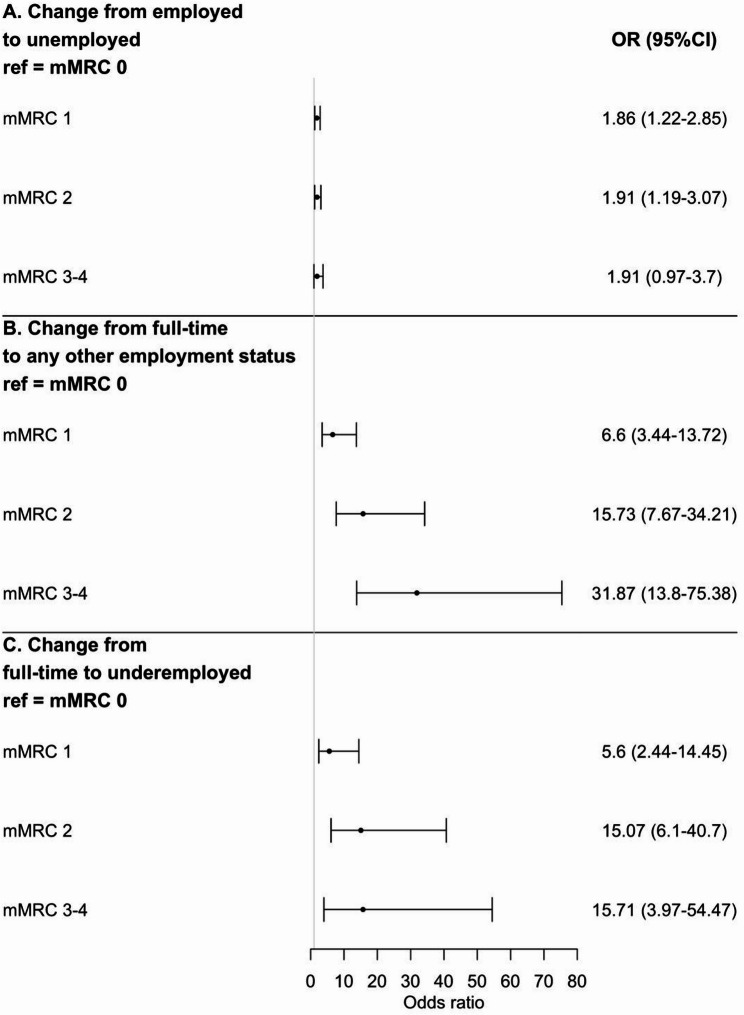
Association between breathlessness and change from employed to unemployed (**A**) and from full-time to any other employment status to assess underemployment. Logistic regression adjusted for age, sex, BMI and rurality. Employed = “Employed full-time”, “Employed part-time”, “Employed casual”, “Self-employed”, “On paid leave”. Unemployed = “On unpaid leave”, “Retired” or “Other”. Underemployed = being employed but not answering “Employed full-time”. Abbreviations: CI = confidence intervals; mMRC = modified Medical Research council breathlessness scale; OR = Odds ratio.

Breathlessness was associated with changing from employed to unemployed, and similar strength of association could be seen in mMRC1 OR: 1.86; 1.22–2.85; mMRC2 OR: 1.91, 1.19–3.07, and mMRC3-4 OR: 1.91; 0.97–3.70. Figure 2a).

Breathlessness was strongly associated with changing from full-time employment to any other employment status and the association strengthens with greater severity: mMRC1 OR: 6.60; 3.44, 13.72; mMRC2 OR: 15.73; 7.67–34.21; and mMRC3-4 OR: 31.87; 13.80–75.38; (Fig. 2b).

Breathlessness was also strongly associated with changing from full-time to underemployed, and the strength increased with each mMRC grade: mMRC 1 OR: 5.60; 2.44–14.45, mMRC 2 OR: 15.07; 6.10–40.70, mMRC 3–4 OR: 15.71; 3.97–54.47, Fig. 2c. However, the confidence intervals for changing from full-time employment to any other employment or to underemployed were broad, indicating insecure results possibly due to too few respondents within this category.

## Discussion

### Main findings

The main findings in this study are that breathlessness was associated with changes in employment status. Even low levels of reported exertional breathlessness (mMRC 1) were significantly associated with unemployment, with higher levels of breathlessness demonstrating stronger relationships. More severe self-reported breathlessness was also associated with a higher probability of working less than full-time as indicated in changes from full-time to other employment status.

The findings indicate that breathlessness can impact the working lives of people living with this symptom in different ways. The consequences can be severe such as losing employment due to an inability to manage the symptom or more subtle, such as adjusting one’s level of employment to accommodate the symptom. Importantly, these changes may be unforeseen by the person at the time of their diagnosis and thus unexpected as breathlessness becomes more intense.

Clinicians can play a critical role in raising awareness of these issues by checking whether the person is contemplating a change in employment because of their breathlessness, leveraging that as an opportunity to optimize their symptom management and, by doing so, their capacity to maintain their employment (including level of employment).

This study complements findings of the Birmingham COPD Cohort study which reported that only the level of breathlessness (assessed by the BODE index[Bodymass index, airflow Obstruction, Dyspnea and Exercise capacity index]) was significantly associated with employment (compared to all other measures [age, educational level, history of occupational exposure]) [28]. The findings also align with those that people with COPD (one of the most commonly associated conditions with breathlessness) are at a higher risk of reduced workforce participation [29]. The employment-related losses in this population costed as lost time from work, lifetime losses and premature retirement are significant [30].

### Strengths and limitations

Strengths of this study include the high number of respondents reflecting the whole population in Australia which aids generalizability of the findings.

A limitation is that the study is its cross-sectional design which can only show relations and not establish causality. In this case the relationships we have shown probably, to some degree, goes both ways since breathlessness will impact negatively on the ability to work but the relationship will also partly reflect some of the many known negative health implications caused by being under- or unemployed [5]. Previous longitudinal studies suggest that poorer health often precedes unemployment rather than results from it, indicating that health selection (in our case breathlessness) may explain an important part of the association between employment status and breathlessness [31]. This may support the interpretation that breathlessness, as a marker of impaired health, may contribute to reduced work ability and changes in employment status, although bidirectional effects are likely [31].

A further limitation is that respondents were asked to focus on one change in their employment status. Individuals having several minor changes to their employment status might not have selected the change as a whole, thereby underestimating the total effect. The current analysis did not include “duration of breathlessness”. The duration might be important because the longer an individual experiences breathlessness the larger the effect on employment status might become.

The odds ratios for employment change increased markedly with higher mMRC grades, but the estimates for the highest mMRC categories were accompanied by wide confidence intervals. This likely reflects the relatively small number of respondents with severe breathlessness and consequently limited statistical precision. These findings should therefore be interpreted with caution, although the overall pattern of increasing odds across mMRC grades remained consistent.

The corona virus pandemic was ongoing during the time of the study which also might influence the results in such a way that breathlessness and other respiratory symptoms might be more in the focus of the general population. It is also possible that some respondents could have interpreted the questions in this survey as pertaining to corona virus infection and change in employment status due to being infected or due to employment threats and losses due to the pandemic and societal lockdowns rather than due to individual inability to work due to exertional breathlessness.

### Implications

Our findings indicate that workplace interventions might be necessary to enable individuals experiencing breathlessness to stay in full-time employment for longer. This may include systematic access to suitable adaptions of the workplace aimed at individuals with breathlessness. People working in physically demanding roles are likely to be particularly disadvantaged. Tailored support may be necessary to support people who need to transition from physical work to less physically demanding employment without having to reduce their employment or move to early retirement. Additional challenges may be related to breathlessness impacting speech and therefore the ability of a person to engage and sustain prolonged conversation [32]. Attention to, and interventions aimed at breathlessness as a cause for shift in employment status might also keep individuals within the workforce for longer, with great value for both the individuals and for society.

Furthermore, our findings reinforce the need for health care professionals to identify breathlessness in patients and investigate its underlying conditions in order to optimize their management, reduce breathlessness intensity and introduce tailored interventions aimed at maintaining work ability.

Our study has focused on self-reported changes in employment status among individuals with breathlessness; however, it is plausible that the impacts of breathlessness extend to other aspects of employment. These may include decreased productivity (also called presenteeism) as well as increased absenteeism from the workplace even among those still in full-time employment [33, 34]. These aspects as well as the impacts of those on productivity valuation and promotional prospects should be assessed in future studies to help obtain a more robust understanding of the consequences of exertional breathlessness on work outcomes for people living with this symptom.

## Conclusion

Experiencing breathlessness is associated with a lower rate of employment as well as a higher probability of reducing work hours and becoming unemployed. This group of individuals may need increased support.

## Supplementary Information

Supplementary Material 1.

## Data Availability

Data sharing statement: All data generated or analysed during this study are included in this published article The study questionnaire is in the public domain and can be accessed at https://osf.io/zwgfc/.

## Abbreviations

ARIA: Accessibility and Remoteness Index of Australia
AU$: Australian Dollar
BMI: Body Mass Index
BODE: Body mass index, airflow Obstruction, Dyspnea, and Exercise capacity index
CI: Confidence Interval
COPD: Chronic Obstructive Pulmonary Disease
DAG: Directed Acyclic Graph
IQR: Interquartile Range
mMRC: Modified Medical Research Council breathlessness scale
OR: Odds Ratio
SD: Standard Deviation
