# Supplementary material for: Breathlessness limiting exertion and under- and unemployment: results from a national, online population survey
Source: BMC Public Health. 2026 Aug 14;26:2356. doi: 10.1186/s12889-026-28996-5 (PMC13474475; doi:10.1186/s12889-026-28996-5)
Supplement: Supplementary file 1 [file 12889_2026_28996_MOESM1_ESM.docx]

| **Question** | **Response options** | **Related categories** |
| --- | --- | --- |
| What is your current employment status? | Employed full-time (1)  Employed part-time (2)  Employed casual (3)  Self-employed (4)  Retired (5)  Unemployed (6)  On paid leave (7)  On unpaid leave (8)  Other (please specify) (9)  Prefer not to say (10)  Not applicable (11) | **Employed** = "Employed full-time", "Employed part-time", "Employed casual", "Self-employed", or "On paid leave".  **Unemployed = “**Unemployed”, “On unpaid leave”, "Retired", or “Other”.  **Underemployed** = Not responding “Employed full-time”  **Missing** = “Prefer not to say” or  “Not applicable” |
| Has your employment status been affected because of your breathlessness? | Yes (1)  No (2)  Prefer not to say (3)  Not applicable (4) | **Missing** = “Prefer not to say” or  “Not applicable” |
| If yes, what was your previous employment status? | Employed full-time (1)  Employed part-time (2)  Employed casual (3)  Self-employed (4)  Other (please specify) (5)  Prefer not to say (6)  Not applicable (7) | - |

**Table S1. Response options and categorisation for employment status.**

**Table S2. Breathlessness severity by employment status.**

|  | **mMRC 0** | **mMRC 1** | **mMRC 2** | **mMRC 3-4** |
| --- | --- | --- | --- | --- |
| **n** | 4593 | 2501 | 685 | 221 |
| **Employment status (%)** |  |  |  |  |
| Employed casual | 338 (7.4) | 198 (7.9) | 50 (7.3) | 11 (5.0) |
| Employed full-time | 2018 (43.9) | 953 (38.1) | 261 (38.1) | 75 (33.9) |
| Employed part-time | 732 (15.9) | 444 (17.8) | 118 (17.2) | 39 (17.6) |
| Not applicable | 53 (1.2) | 24 (1.0) | 9 (1.3) | 6 (2.7) |
| On paid leave | 41 (0.9) | 23 (0.9) | 4 (0.6) | 0 (0.0) |
| On unpaid leave | 29 (0.6) | 22 (0.9) | 7 (1.0) | 2 (0.9) |
| Other | 231 (5.0) | 182 (7.3) | 63 (9.2) | 10 (4.5) |
| Prefer not to say | 32 (0.7) | 17 (0.7) | 9 (1.3) | 5 (2.3) |
| Retired | 282 (6.1) | 128 (5.1) | 46 (6.7) | 32 (14.5) |
| Self-employed | 247 (5.4) | 139 (5.6) | 26 (3.8) | 13 (5.9) |
| Unemployed | 590 (12.8) | 371 (14.8) | 92 (13.4) | 28 (12.7) |
| **Employment affected by breathlessness (%)** | 276 (6.0) | 312 (12.5) | 200 (29.2) | 68 (30.8) |
| **Change from employed to unemployed (%)** | 56 (1.2) | 110 (4.4) | 72 (10.5) | 25 (11.3) |
